# Impact of cardiometabolic index on long-term mortality in young adults with type 2 diabetes mellitus

**DOI:** 10.1371/journal.pone.0348952

**Published:** 2026-05-21

**Authors:** Aihua Li, Ziying Zhou, Xiaoli Chen, Yubo Liu, Qilin Ma

**Affiliations:** 1 Department of Rheumatology and Immunology, Xiangya Hospital, Central South University, Changsha, Hunan, China; 2 National Clinical Research Center for Geriatric Diseases, Xiangya Hospital, Changsha, Hunan, China; 3 Department of Cardiovascular Medicine, Xiangya Hospital, Central South University, Changsha, Hunan, China; Saud Al-Babtain Cardiac Centre, SAUDI ARABIA

## Abstract

**Background:**

The cardiometabolic index (CMI) has been utilized in recent years to detect patients with type 2 diabetes mellitus (T2DM). Given the consistent annual rise in the incidence of T2DM among young adults, the relationship between CMI and mortality risk among those with early-onset T2DM remains to be determined.

**Methods:**

We enrolled 2,188 participants aged 20–65 with diabetes from the National Health and Nutrition Examination Survey (NHANES) 1999–2018. Weighted Cox proportional hazard regression models were employed to evaluate hazard ratios (HRs) with 95% confidence intervals (CIs). Restricted cubic spline (RCS) curves were utilized to assess linear associations. Stratified and interaction analyses were performed.

**Results:**

We find that higher CMI is strongly associated with an increased risk of all-cause mortality (*p* = 0.005) and cardiovascular mortality (*p* = 0.020) in the strictest model. RCS analysis revealed a linear relationship between CMI and mortality. Subgroup and interaction analysis showed no statistical significance.

**Conclusions:**

The higher CMI level is correlated with all-cause and cardiovascular mortality risk among young individuals with diabetes mellitus, and serves as a comprehensive and effective prognostic indicator for long-term health.

## Introduction

In recent years, the prevalence of diabetes mellitus (DM) has continued rising globally, presenting a formidable public health concern. According to estimates from the International Diabetes Federation (IDF), DM affected 536.6 million people, representing 10.5% of the population worldwide in 2021. Projections suggest that by 2045 this number will reach 783.2 million, accounting for 12.2% of the global population [1]. The Global Burden of Disease Study 2021 estimated that diabetes was responsible for as many as 1.7 million deaths globally in 2021 (1.57–1.79 million) [2]. Cardiovascular complications, including peripheral artery disease (PAD), coronary artery disease (CAD), heart failure (HF), and stroke, remain the leading causes of morbidity and mortality among individuals with DM [3]. These conditions are twice as prevalent in individuals with type 2 diabetes mellitus (T2DM) compared to their counterparts without the disease [4]. With the decreasing onset age of T2DM, its prevalence among young adults is rising steadily each year [5]. Early-onset T2DM exposes patients to prolonged metabolic derangements, which accelerate and hasten the progression of atherosclerosis, heart failure, and other cardiovascular conditions. Several studies have demonstrated that patients with young-onset type 2 diabetes (YOD) face a significantly greater risk of macrovascular complications compared to those with late-onset type 2 diabetes (LOD) or type 1 diabetes, as well as elevated mortality rates from all-causes and cardiovascular disease (CVD) [6–8]. The rising incidence of YOD presents a significant clinical challenge. Thus, identifying reliable biomarkers in young individuals with diabetes is crucial for reducing mortality rates and enhancing patient prognosis.

Patients with DM are often predisposed to obesity and dyslipidemia, which can further accelerate the progression of CVD. The Cardiometabolic Index (CMI) has recently emerged as an innovative marker for comprehensively assessing cardiometabolic risk by integrating two key indicators—waist-to-height ratio (WHtR) and triglycerides-to-high-density lipoprotein cholesterol (TG/HDL-C) ratio—which reflect central adiposity and lipid metabolism, both of which are closely linked to cardiovascular health [9,10]. CMI has been evaluated as a predictive marker across various conditions. For instance, it has been shown to be effective in identifying the existence and intensity of metabolic syndrome in adults with obesity [11]. Additionally, emerging research highlights CMI’s association with metabolic-associated fatty liver disease (MAFLD) [12] and hypertension [13]. Furthermore, elevated CMI has been positively correlated with all-cause mortality in the elderly, suggesting that it may function as a valuable prognostic indicator for poor outcomes in this population. Studies have demonstrated a significant relationship between elevated CMI and the development of diabetes [14], as well as its association with arteriosclerosis in patients with T2DM, further highlighting its value as a cardiovascular risk marker [15]. However, while CMI has been studied with diabetes, research on its potential as an indicator of all-cause and cardiovascular mortality, particularly among younger diabetic populations, remains limited. Given the growing prevalence of young-onset diabetes and the heightened cardiovascular risk in this group, it is crucial to explore whether CMI can function as a reliable predictor of mortality.

In this study, we utilized National Health and Nutrition Examination Survey (NHANES) data from younger diabetic patients to investigate the correlation between CMI and both all-cause and cardiovascular mortality. Our analysis aims to determine whether CMI serves as a reliable predictor of mortality in this population, offering a potential tool for risk stratification and personalized management in young diabetic patients.

## Materials and methods

### Source of data and study participants

**Fig 1:**
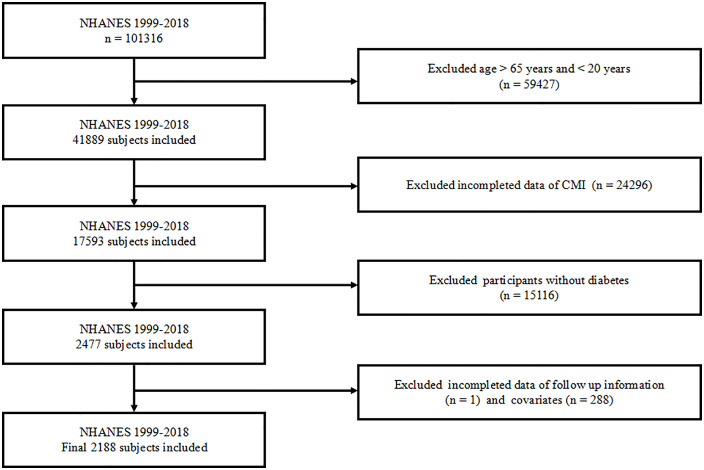
Participants flowchart. Abbreviation: NHANES: National Health and Nutrition Examination Survey; CMI: cardiometabolic index.

The data for this prospective cohort study were collected from NHANES between 1999–2018 administered by the National Center for Health Statistics (NCHS) and the Centers for Disease Control and Prevention (CDC). The participants underwent comprehensive interviews, physical examinations, and laboratory assessments in specialized mobile centers, administered by skilled and experienced professionals and technical staff. This study encompassed participants (n = 101,316) from the NHANES 1999–2018 cohort, subsequently screening those aged 20–65 years who had available CMI levels. Subsequently, DM participants with missing data of follow-up information and any covariates in this study were excluded, resulting in a final sample size of 2188 individuals (Fig 1). Each participant provided written informed consent for participation in the program, thus additional written consent was not required for further analysis.

### Determination of all-cause and cardiovascular mortality

In this study, we assessed outcomes encompassing both all-cause mortality and CVD mortality. Data on all-cause mortality were obtained by probabilistically linking NHANES records to the National Death Index (NDI) documentation. Supplementary mortality surveillance data were sourced from the US Social Security Administration, the Centers for Medicare and Medicaid Services, and official death certificates. The cause of death was classified using standardized codes from the International Classification of Diseases, Tenth Revision (ICD-10). Mortality attributable to heart disease including coronary heart disease (CHD), angina, congestive heart failure (CHF), cardiac arrest, and stroke was identified by ICD-10 codes I00-I09, I11, I13, and I20–I51. The follow-up period was defined as the interval extending from the date of the examination to either the date of death or the end of the follow-up period, which concluded on December 31, 2019.

### Definition of diabetes

Diabetes was diagnosed based on one or more of the following criteria: i. fasting plasma glucose (FPG) levels ≥7 mmol/L (126 mg/dL); ii. glycated hemoglobin A1c (HbA1c) ≥6.5%; iii. a 2-hour blood glucose level ≥200 mg/dL from an oral glucose tolerance test (OGTT). Additionally, a self-reported diagnosis or the use of insulin or oral hypoglycemic agents was also considered indicative of diabetes [16].

### Assessment of the Cardiometabolic Index

To compute the CMI, the following formula was employed: CMI= (TG÷HDL−C)×WHtR, where TG refers to triglycerides, HDL-C denotes high-density lipoprotein cholesterol, and WHtR represents the waist circumference to height ratio. In our study, CMI was treated as an exposure variable, and all participants were stratified into high or low CMI groups based on their CMI values for subsequent analyses

### Covariates

In the present study, we considered essential covariates including the following: age, sex (female/male), race (Mexican American/Non-Hispanic Black/Non-Hispanic White/Other Hispanic/ Others), education levels (under high school/ high school or equivalent/above high school), smoking status (never/former/current), drinking status (no/low-to-moderate/heavy), body mass index (BMI), waist circumference, WHtR, total cholesterol (TC), TG, estimated glomerular filtration rate (eGFR), low-density lipoprotein cholesterol (LDL), HDL, and disease status (hypertension/CVD). BMI was calculated by dividing weight (measured in kilograms) by the square of height (expressed in meters). Participants were categorized into categories of normal weight (BMI < 25 kg/m²), overweight (BMI ranging from 25 to 30 kg/m²), or obese (BMI ≥ 30 kg/m²). Hypertension was defined by the following clinical criteria: three consecutive measurements of systolic blood pressure surpassing 140 mmHg or diastolic blood pressure exceeding 90 mmHg, a clinical diagnosis of hypertension, or employment of antihypertensive pharmaceuticals.

### Statistical analysis

All statistical analyses took into account the complex survey design of NHANES, incorporating sample weights, clustering, and stratification. In this study, participant characteristics at baseline were presented as means with standard deviations (SD) for continuous variables, and as counts with weighted percentages for categorical variables. The enrolled participants were stratified into two categories according to the optimal cutoff of the CMI and intergroup analyses were performed. Where applicable, weighted chi-square tests and Kruskal-Wallis tests were utilized to assess the distribution of fundamental characteristics between the different CMI groups. The survey-weighted univariable and multivariable Cox proportional hazards regression models were employed to evaluate the hazard ratios (HRs) with 95% confidence intervals (CIs) for all-cause and CVD risk concerning CMI. Three models were employed, model 1 adjusted none; model 2 adjusted by age, sex, and ethnicity; model 3 additionally adjusted by education level, smoking status, drinking status, weight, TC, eGFR, hypertension and CVD based on model 2. Kaplan-Meier curves and inter-group comparisons by log-rank tests demonstrated survival outcomes. Restricted cubic spline (RCS) analysis was employed to examine the potential nonlinear relationships. Stratified and interaction analyses concerned covariates contained age, sex, ethnicity, education level, smoking status, drinking status, weight, TC, eGFR, hypertension and CVD. In addition, Spearman correlation coefficients were calculated to assess the correlations among CMI and its components and metabolic markers (fasting glucose, HbA1c, fasting insulin). Receiver operating characteristic (ROC) curves and the area under the curve (AUC) were employed to predict the performance of CMI, its components (TG/HDL, WHtR), and metabolic markers (fasting glucose, HbA1c, fasting insulin) for all-cause events and CVD events. Differences in AUC between CMI and comparator markers were tested using DeLong’s test (for correlated ROC curves). All statistical analyses were employed by R (version 4.3.1), with a two-tailed p-value of less than 0.05 regarded as statistically significant.

## Results

### Baseline Characteristics

**Fig 2:**
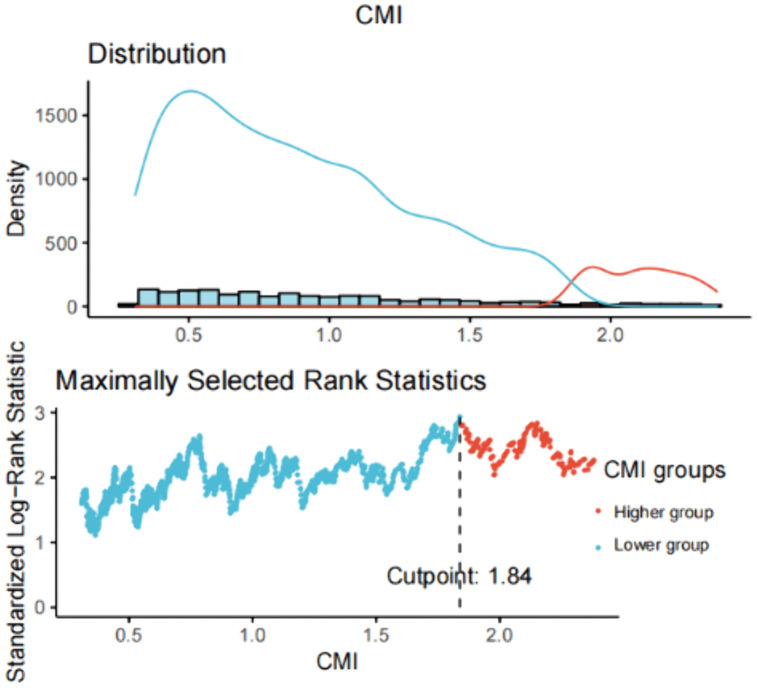
Cut-off point determination for the cardiometabolic index. The cut-off point of CMI was calculated using the maximally selected rank statistics based on the ‘maxstat’ package. Abbreviation: CMI: cardiometabolic index.

This study included 2,188 participants from a young diabetic population, representing an estimated 17,955,290 young individuals with diabetes across the United States. Using an optimal CMI cutoff of 1.84, determined by maximally selected rank statistics for the strongest survival association, participants were divided into two groups: a higher CMI group (CMI > 1.84, n = 366) and a lower CMI group (CMI ≤ 1.84, n = 1822) (Fig 2). Compared to the lower CMI group, participants in the higher CMI group were predominantly male, had a greater proportion of Mexican Americans, and exhibited lower LDL and HDL levels, alongside higher BMI, WC, WHtR, TC, TG, HbA1c, and fasting glucose levels. Additionally, this group had a higher prevalence of hypertension and CVD. Further characteristics of the participants are detailed in Table 1.

**Table 1:** Weighted baseline characteristics of included participants according to the CMI.

Characteristic	All participants (n = 2188)	Lower CMI (n = 1822)	Higher CMI (n = 366)	*p-*value
**Age, years**	51.10 (10.53)	51.30 (10.45)	50.10 (10.91)	0.3
**Sex**				<0.001
male	1,132 (53.0%)	910 (51.0%)	222 (63.3%)	
female	1,056 (47.0%)	912 (49.0%)	144 (36.7%)	
**Race**				0.004
Mexican American	503 (11.4%)	392 (10.6%)	111 (15.6%)	
Non-Hispanic Black	531 (14.9%)	483 (16.1%)	48 (8.6%)	
Non-Hispanic White	654 (57.6%)	523 (57.2%)	131 (59.8%)	
Others	500 (16.1%)	424 (16.1%)	76 (16%)	
**Education levels**				0.5
Under high school	350 (8.8%)	278 (8.5%)	72 (10.7%)	
High school or equivalent	869 (39.9%)	726 (40.2%)	143 (38.5%)	
Above high school	969 (51.2%)	818 (51.3%)	151 (50.8%)	
**Smoking status**				0.15
Never smoker	1,108 (49.5%)	952 (50.7%)	156 (43.5%)	
Former smoker	587 (28.0%)	476 (27.7%)	111 (29.4%)	
Current smoker	493 (22.5%)	394 (21.6%)	99 (27.2%)	
**Drinking status**				0.6
Nondrinker	916 (37.7%)	757 (37.5%)	159 (38.6%)	
Low-to-moderate drinker	616 (30.7%)	527 (31.2%)	89 (28.0%)	
Heavy drinker	656 (31.6%)	538 (31.3%)	118 (33.4%)	
**BMI, kg/m** ^**2**^	33.57 (7.86)	33.14 (7.96)	35.76 (6.93)	<0.001
**BMI category**				<0.001
Normal weight (<25)	269 (11.8%)	254 (13.7%)	15 (2.0%)	
Overweight (25–30)	614 (24.7%)	522 (25.2%)	92 (22.0%)	
Obesity (≥30)	1,305 (63.5%)	1,046 (61.1%)	259 (75.9%)	
**WC, cm**	111.41 (17.53)	110.12 (17.59)	118.04 (15.62)	<0.001
**WHtR**	0.66 (0.10)	0.65 (0.10)	0.70 (0.09)	<0.001
**TC, mmol/L**	5.03 (1.21)	4.91 (1.11)	5.62 (1.47)	<0.001
**TG, mmol/L**	2.08 (2.38)	1.51 (0.66)	5.05 (4.72)	<0.001
**LDL, mmol/L**	2.92 (0.96)	2.93 (0.96)	2.79 (0.94)	0.063
**HDL, mmol/L**	1.23 (0.36)	1.29 (0.34)	0.90 (0.19)	<0.001
**HbA1c, %**	7.16 (1.84)	7.00 (1.73)	8.02 (2.11)	<0.001
**Fast glucose, mg/dl**	155.07 (63.88)	148.30 (56.93)	190.06 (83.54)	<0.001
**eGFR, ml/min/1.73m** ^**2**^	96.73 (20.39)	96.41 (20.23)	98.37 (21.12)	0.095
**Hypertension**	1,379 (61.8%)	1,132 (60.5%)	247 (69.0%)	0.051
**CVD**	360 (16.0%)	292 (15.1%)	68 (21.2%)	0.022
**CMI**	1.36 (1.96)	0.84 (0.44)	4.04 (3.76)	<0.001

CMI: cardiometabolic index; BMI: body mass index; WC waist circumference; WHtR: waist-to-height ratio; TC: total cholesterol; TG: triglyceride; LDL: low-density lipoprotein cholesterol; HDL: high-density lipoprotein cholesterol; HbA1c: glycated hemoglobin A1c; eGFR: estimated glomerular filtration rate; CVD: cardiovascular disease. Data are presented as mean (SD) or n (%);

### Associations of the CMI with all‑cause and cardiovascular mortality

**Fig 3:**
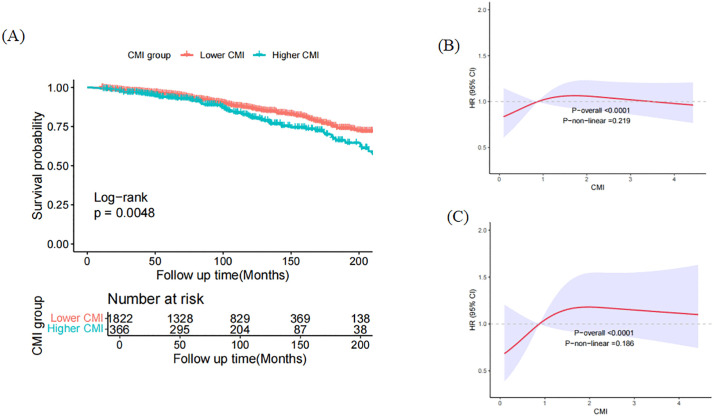
Kaplan-Meier survival curves and restricted cubic spline (RCS) analysis for the association between CMI and mortality risk. Kaplan–Meier analysis of all-cause mortality (A) based on CMI groups among young individuals with diabetes; Restricted cubic spline analysis between CMI and the risk of all-cause mortality (B) and CVD mortality (C) in young participants with diabetes. Abbreviation: CMI: cardiometabolic index; CVD: cardiovascular disease.

Over a mean follow-up duration of 8.62 years, 294 of the 2188 young diabetic patients (13.4%) succumbed. Of these, 84 (3.84%) were attributed to cardiovascular causes. Using a weighted multivariable Cox regression model, we examined the correlation between CMI and all-cause and cardiovascular mortality. In the unadjusted analysis, individuals with higher CMI had a significantly elevated risk of all-cause mortality (HR = 1.91, *p* < 0.001) and cardiovascular mortality (HR = 2.28, *p* = 0.002). The association remained robust after multivariate adjustments in both Model 2 (HR = 1.96, *p* < 0.001) and Model 3 (HR = 1.67, *p* = 0.005) for all-cause mortality and (HR = 2.34, *p* = 0.002) (HR = 2.03, *p* = 0.020) for cardiovascular mortality (Table 2). In addition, Kaplan‒Meier survival analysis revealed notable disparities in all-cause mortality rates between the groups with higher and lower CMI levels (*p* = 0.0048), with the higher-CMI cohort exhibiting a notably reduced survival rate (Fig 3A). RCS analysis revealed a linear relationship between CMI and all-cause (*p* for non-linearity = 0.219) and cardiovascular mortality (*p* for non-linearity = 0.186) (Fig 3B and 3C). Furthermore, the Spearman correlation coefficients between CMI and TG/HDL revealed a high correlation (0.99) (S1 Fig.). RCS analysis revealed a non-linear relationship between TG/HDL, WHtR, glucose and all-cause and cardiovascular mortality (all *p* for non-linearity < 0.05), and a linear relationship between HbA1c, fast insulin and all-cause mortality (all p for non-linearity > 0.05) (S2 Fig.). Moreover, after adjustment, the association between glucose, HbA1c, fast insulin and all-cause mortality was statistically significant (HR = 1.004, *p* < 0.001; HR = 1.142, *p* < 0.001; HR = 1.003, *p* = 0.033), and the association between glucose and CVD mortality was statistically significant (HR = 1.003, *p* = 0.003). The association between WHtR, TG/HDL and all-cause mortality and CVD mortality was not statistically significant (S1 Table). In addition, the AUC of CMI and the other biomarkers in all-cause mortality and CVD mortality were demonstrated in S3 Fig. and S2 Table.

**Table 2:** The association of CMI with all-cause and cardiovascular mortality in young diabetes population.

Characteristic	Model 1		Model 2		Model 3	
	HR (95% CI)	*p*-value	HR (95% CI)	*p*-value	HR (95% CI)	*p*-value
All-cause mortality						
CMI category						
Lower CMI	Ref		Ref		Ref	
Higher CMI	1.91 (1.40, 2.60)	<0.001	1.96 (1.43, 2.68)	<0.001	1.67 (1.17, 2.37)	0.005
Cardiovascular mortality						
CMI category						
Lower CMI	Ref		Ref		Ref	
Higher CMI	2.28 (1.34, 3.86)	0.002	2.34 (1.37, 3.98)	0.002	2.03 (1.12, 3.71)	0.020

Model 1: No covariates were adjusted

Model 2: Age, gender, and race were adjusted

Model 3: Age, gender, race, education level, smoking status, drinking status, weight, TC, eGFR, hypertension and CVD were adjusted

CMI: cardiometabolic index; TC: total cholesterol; eGFR: estimated glomerular filtration rate; HR, Hazard Ratio 95%CI, 95% Confidence interval.

### Subgroup analysis

**Fig 4:**
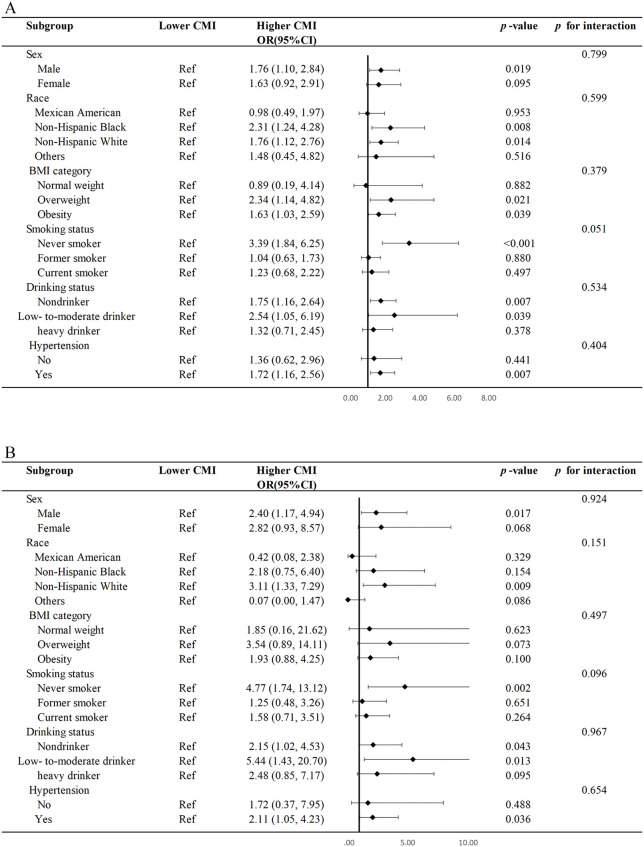
Stratified analyses of the associations between CMI and mortality outcomes. Stratified analyses of the associations between CMI and all-cause mortality and CVD mortality among young individuals with diabetes. Abbreviation: CMI: cardiometabolic index; CVD: cardiovascular disease.

Subgroup analyses were conducted to assess the associations between CMI and all-cause and cardiovascular mortality (Fig 4). The results found that being male, obese, never smoking, a non-drinker or low-to-moderate alcohol drinker, and having hypertension were more strongly associated with an increased risk of all-cause mortality. Furthermore, being male, never smoking, being a low-to-moderate alcohol drinker, and having hypertension were more strongly associated with an elevated risk of cardiovascular mortality. Importantly, no significant interactions were detected between CMI and any of the stratification variables.

## Discussion

In the present study, we explored the association of CMI with CVD and mortality in a cohort of young individuals with diabetes, utilizing NHANES data spanning from 1999 to 2018. Our findings indicate that a higher CMI is strongly associated with an increased risk of all-cause and cardiovascular mortality, even after adjusting for multiple confounding factors. The linear relationship between CMI and mortality, as revealed by RCS analysis, suggests that even modest increases in CMI are associated with heightened mortality risk. Subgroup analysis uncovered that the relationship between elevated CMI and mortality risk was stronger among male participants, individuals with obesity, non-smokers, and those with hypertension. The Cox regression results and AUC results confirm that CMI outperforms its anthropometric component (WHtR) and insulin, and is non-inferior to glucose/HbA1c, supporting its utility as a composite cardiometabolic marker. The statistically significant relationships suggest that CMI may be a valuable relevant marker for overall mortality even specifically for cardiovascular outcomes in this population.

The global of young individuals with T2DM was increasing annually. High energy intake, obesity, a sedentary lifestyle, and decreased moderate to high-intensity training led to the rising tendency [17]. Reduced physical activity and a sedentary lifestyle are correlated with an elevated risk of long-term health outcomes, including all-cause mortality and the incidence of cardiovascular disease in individuals with T2DM [18]. This decline in physical engagement may lead to increased insulin resistance and the promotion of low-grade systemic inflammation, both of which could significantly contribute to these adverse health outcomes [19–21]. Current evidence suggests a heightened risk of cardiovascular complications, adverse pregnancy outcomes, and premature mortality among young individuals with T2DM [22–24]. The precise mechanisms by which diabetes contributes to mortality remain shrouded in uncertainty, however, insulin resistance may play a pivotal role in diabetic individuals’ fatality. Insulin resistance is a pathophysiological condition defined as the diminished ability of insulin to optimally facilitate the transport of glucose into bodily cells, which results in reduced insulin bioavailability and impaired glucose uptake, ultimately leading to hyperinsulinemia [25]. Consequently, hyperinsulinemia further triggers inflammatory responses, evidenced by elevated levels of pro-inflammatory cytokines such as nuclear factor kappa B (NF-κB), tumor necrosis factor-alpha (TNF-α), interleukin-6 (IL-6), and interleukin-8 (IL-8) [26–28]. Moreover, insulin enhances the production of endothelial nitric oxide by activating the phosphatidylinositol 3-kinase pathway, thereby inducing vasodilation. In states of insulin resistance, this pathway is compromised, leading to diminished nitric oxide levels and subsequent stimulation of the mitogen-activated protein kinase pathway, which facilitates vasoconstriction [29]. The production of these factors subsequently promotes smooth muscle cell proliferation and collagen deposition, ultimately resulting in vascular arterial stiffness, which promotes the development of cardiovascular disease [25,30]. It is consequential to employ a predictive metric to ascertain the all-cause even cardiovascular risk for these young diabetics, thereby mitigating the likelihood of early-onset complications.

The CMI consists of the waist-to-height ratio and lipid profiles as triglycerides-to-HDL cholesterol ratio, which was initially innovated to identify diabetes mellitus. The waist-to-height ratio serves as a more effective screening instrument compared to waist circumference and BMI for assessing cardiometabolic risk factors in adults [9]. The ratio of triglycerides to HDL cholesterol functions effectively as a screening tool for detecting impaired glucose tolerance [31], the higher ratio correlated with elevated insulin resistance and increased risk of coronary heart disease (CHD), cardiovascular disease (CVD) in obese patients with T2DM [32]. CMI comprehensively presents central adiposity and lipid metabolism, tightly associated with cardiovascular health [14]. A national cohort study demonstrated elevated levels of CMI with a remarkably elevated risk of new-onset T2DM (HR = 1.78) in middle-aged and elderly, as those with lower levels of CMI changed to higher levels during follow-up time with the risk of T2DM increased by 75% [33]. Moreover, CMI is not only correlated to elevated risk of T2DM but also tightly linked with other metabolic diseases. The positive correlation found in CMI with systemic inflammatory status among the obesity group contrasts with the normal weight group in older men (*P* < 0.0001) [34]. Consistent investigation was reported by Xu et al. that CMI was positively associated with long-term health outcomes as all-cause mortality (HR = 1.11) in the elderly, additionally, inflammation indicators such as leukocytes and neutrophils mediated the association [35]. Zou et al. investigated the CMI-related risk of non-alcoholic fatty liver disease (NAFLD) among young individuals (OR = 2.63) considerably higher contrasts with young and middle-aged (OR = 1.38), middle-aged and elderly (OR = 1.18), and elderly (OR = 1.14) [36]. Population-based cohort studies showed that the lower CMI group was younger than the higher group. After strict adjustment, the significant positive correlation remained to verify the comprehensiveness and applicability of CMI [36,37]. Our findings suggest that CMI may be particularly useful for identifying at-risk individuals who were obese, non-smokers, or had hypertension inspired by subgroup analysis, further emphasizing the importance of personalized risk assessment in clinical practice and evaluating long-term health for individuals. Massive studies emphasized that increased CMI levels are related to deteriorated prognosis, the potential mechanisms need to be discovered by further advanced research.

Given the high correlation between CMI and the triglyceride-to-HDL cholesterol (TG/HDL-C) ratio observed in our study, and their comparable AUC values for mortality prediction, a legitimate question arises regarding the practical advantage of the more complex CMI formula. We acknowledge that TG/HDL-C is a simpler and well-established surrogate for insulin resistance. Nevertheless, CMI offers several distinct advantages from a clinical and pathophysiological perspective. First, CMI explicitly incorporates waist-to-height ratio (WHtR), a direct measure of central adiposity that is independent of lipid metabolism. Central obesity is not merely a proxy for dyslipidemia; it contributes to mortality through adipokine dysregulation, chronic low-grade inflammation, and mechanical effects on cardiovascular function—mechanisms that are incompletely captured by TG/HDL-C alone. For instance, visceral fat accumulation drives adipokine dysregulation, which directly impairs metabolic homeostasis and promotes insulin resistance [38,39]. Moreover, central obesity is a state of chronic low-grade inflammation. Visceral adipose tissue serves as an active compartment for secreting pro-inflammatory molecules [40]. Furthermore, central obesity exerts direct mechanical effects on the cardiovascular system. Excess visceral fat increases total peripheral resistance and cardiac workload, promotes adverse cardiac remodeling, and accelerates arterial stiffening independent of metabolic factors [41–43]. In young individuals with type 2 diabetes, visceral adiposity often precedes overt dyslipidemia, and an isolated elevation in WHtR with normal TG/HDL-C may still confer excess risk [44–46]. Second, our subgroup analyses revealed that CMI exhibited stronger associations with mortality in certain populations (e.g., non-smokers, hypertensive individuals) compared to TG/HDL-C (S4 Fig.), suggesting that the inclusion of WHtR may capture obesity-related risk that is not fully reflected by lipid ratios in specific clinical contexts [47]. Third, the two-component structure of CMI (WHtR and TG/HDL-C) allows clinicians to disentangle the relative contributions of adiposity versus lipid metabolism to an individua’s risk profile, thereby guiding more targeted interventions—e.g., lifestyle modification for elevated WHtR versus lipid-lowering therapy for elevated TG/HDL-C [12,48,49]. In contrast, TG/HDL-C as a single composite metric provides no such decomposition. Therefore, CMI offers enhanced granularity for personalized risk assessment and mechanistic understanding, particularly in young diabetic patients where central obesity is a dominant and modifiable driver of cardiometabolic risk.

The strengths of this study are the employment of representative and weighted NHANES data for generalizability, and we provide evidence of CMI positivity related to all-cause and cardiovascular mortality in young individuals with diabetes. Our findings also emphasize the clinical implications of CMI. Given the rising prevalence of young-onset diabetes, early identification of individuals at higher risk of mortality is critical for implementing timely interventions. CMI, which is a simple, non-invasive metric derived from commonly measured clinical parameters, could serve as a valuable tool for risk stratification in young diabetic patients. Subsequent research endeavors should focus on confirming these results within broader and more varied demographic groups, as well as investigating the possible mechanisms underlying the differential associations between CMI and mortality outcomes. Additionally, the limitations of this study are the following: firstly, unmeasured confounding factors exist despite adjusting for some covariates; secondly, causality could not be confirmed in this study due to the observational nature of cohort studies.

## Conclusion

In this prospective population-based study, we observed significant findings that the higher CMI level correlated with mortality risk among young individuals with diabetes mellitus and served as a comprehensive and effective prognostic indicator for long-term health.

## Supporting information

S1 Fig The Spearman correlation coefficients among CMI, TG/HDL, WHtR, fast glucose, HbA1c, and fast insulin. (PDF)

S2 Fig The Restricted cubic spline analysis between TG/HDL, WHtR, fast glucose, HbA1c, fast insulin and the risk of all-cause mortality and CVD mortality in participants with diabetes. (PDF)

S3 Fig Receiver operating characteristic (ROC) curves of CMI and other metabolic indicators for predicting mortality risk. (PDF)

S4 Fig Stratified analyses of the associations between TG/HDL and all-cause mortality and CVD mortality among young individuals with diabetes. (PDF)

S1 Table Association between TG/HDL, WHtR, glucose, HbA1c, fast insulin and all-cause mortality and CVD mortality in T2DM populations. (PDF)

S2 Table AUC of TG/HDL, WHtR, glucose, HbA1c, fast insulin and all-cause mortality and CVD mortality in T2DM populations. (PDF)

## References

[1] Sun H, Saeedi P, Karuranga S, Pinkepank M, Ogurtsova K, Duncan BB, et al. IDF Diabetes Atlas: Global, regional and country-level diabetes prevalence estimates for 2021 and projections for 2045. Diabetes Res Clin Pract. 2022;183:109119. doi: 10.1016/j.diabres.2021.109119 34879977 PMC11057359

[2] GBD 2021 Diabetes Collaborators. Global, regional, and national burden of diabetes from 1990 to 2021, with projections of prevalence to 2050: a systematic analysis for the Global Burden of Disease Study 2021. Lancet Lond Engl. 2023;402(10397):203–34. doi: 10.1016/S0140-6736(23)01301-6 PMC10364581 37356446

[3] Zheng Y, Ley SH, Hu FB. Global aetiology and epidemiology of type 2 diabetes mellitus and its complications. Nat Rev Endocrinol. 2018;14(2):88–98. doi: 10.1038/nrendo.2017.151 29219149

[4] Shah AD, Langenberg C, Rapsomaniki E, Denaxas S, Pujades-Rodriguez M, Gale CP, et al. Type 2 diabetes and incidence of cardiovascular diseases: a cohort study in 1·9 million people. Lancet Diabetes Endocrinol. 2015;3(2):105–13. doi: 10.1016/S2213-8587(14)70219-0 25466521 PMC4303913

[5] Magliano DJ, Sacre JW, Harding JL, Gregg EW, Zimmet PZ, Shaw JE. Young-onset type 2 diabetes mellitus - implications for morbidity and mortality. Nat Rev Endocrinol. 2020;16(6):321–31. doi: 10.1038/s41574-020-0334-z 32203408

[6] Huo L, Magliano DJ, Rancière F, Harding JL, Nanayakkara N, Shaw JE, et al. Impact of age at diagnosis and duration of type 2 diabetes on mortality in Australia 1997-2011. Diabetologia. 2018;61(5):1055–63. doi: 10.1007/s00125-018-4544-z 29473119

[7] Constantino MI, Molyneaux L, Limacher-Gisler F, Al-Saeed A, Luo C, Wu T, et al. Long-term complications and mortality in young-onset diabetes: type 2 diabetes is more hazardous and lethal than type 1 diabetes. Diabetes Care. 2013;36(12):3863–9. doi: 10.2337/dc12-2455 23846814 PMC3836093

[8] Hillier TA, Pedula KL. Complications in young adults with early-onset type 2 diabetes: losing the relative protection of youth. Diabetes Care. 2003;26(11):2999–3005. doi: 10.2337/diacare.26.11.2999 14578230

[9] Ashwell M, Gunn P, Gibson S. Waist-to-height ratio is a better screening tool than waist circumference and BMI for adult cardiometabolic risk factors: systematic review and meta-analysis. Obes Rev. 2012;13(3):275–86. doi: 10.1111/j.1467-789X.2011.00952.x 22106927

[10] Kosmas CE, Rodriguez Polanco S, Bousvarou MD, Papakonstantinou EJ, Peña Genao E, Guzman E, et al. The Triglyceride/High-Density Lipoprotein Cholesterol (TG/HDL-C) Ratio as a Risk Marker for Metabolic Syndrome and Cardiovascular Disease. Diagnostics (Basel). 2023;13(5):929. doi: 10.3390/diagnostics13050929 36900073 PMC10001260

[11] Tamini S, Bondesan A, Caroli D, Sartorio A. The Lipid Accumulation Product Index (LAP) and the Cardiometabolic Index (CMI) Are Useful for Predicting the Presence and Severity of Metabolic Syndrome in Adult Patients with Obesity. J Clin Med. 2024;13(10):2843. doi: 10.3390/jcm13102843 38792386 PMC11122168

[12] Duan S, Yang D, Xia H, Ren Z, Chen J, Yao S. Cardiometabolic index: A new predictor for metabolic associated fatty liver disease in Chinese adults. Front Endocrinol (Lausanne). 2022;13:1004855. doi: 10.3389/fendo.2022.1004855 36187093 PMC9523727

[13] Wang H, Chen Y, Sun G, Jia P, Qian H, Sun Y. Validity of cardiometabolic index, lipid accumulation product, and body adiposity index in predicting the risk of hypertension in Chinese population. Postgrad Med. 2018;130(3):325–33. doi: 10.1080/00325481.2018.1444901 29478365

[14] Wakabayashi I, Daimon T. The “cardiometabolic index” as a new marker determined by adiposity and blood lipids for discrimination of diabetes mellitus. Clin Chim Acta. 2015;438:274–8. doi: 10.1016/j.cca.2014.08.042 25199852

[15] Tang C, Pang T, Dang C, Liang H, Wu J, Shen X, et al. Correlation between the cardiometabolic index and arteriosclerosis in patients with type 2 diabetes mellitus. BMC Cardiovasc Disord. 2024;24(1):186. doi: 10.1186/s12872-024-03853-8 38539102 PMC10976822

[16] Zou X, Zhou X, Zhu Z, Ji L. Novel subgroups of patients with adult-onset diabetes in Chinese and US populations. Lancet Diabetes Endocrinol. 2019;7(1):9–11. doi: 10.1016/S2213-8587(18)30316-4 30577891

[17] Lascar N, Brown J, Pattison H, Barnett AH, Bailey CJ, Bellary S. Type 2 diabetes in adolescents and young adults. Lancet Diabetes Endocrinol. 2018;6(1):69–80. doi: 10.1016/S2213-8587(17)30186-9 28847479

[18] Lu J, Cao X, Chang X, Zheng G, Zhu H, Gao S, et al. Associations between physical activity and all-cause and cardiovascular mortality in adults with type 2 diabetes mellitus: A prospective cohort study from NHANES 2007-2018. Prim Care Diabetes. 2024;18(1):44–51. doi: 10.1016/j.pcd.2023.11.010 38052713

[19] Hamburg NM, McMackin CJ, Huang AL, Shenouda SM, Widlansky ME, Schulz E, et al. Physical inactivity rapidly induces insulin resistance and microvascular dysfunction in healthy volunteers. Arterioscler Thromb Vasc Biol. 2007;27(12):2650–6. doi: 10.1161/ATVBAHA.107.153288 17932315 PMC2596308

[20] Mazzucco S, Agostini F, Biolo G. Inactivity-mediated insulin resistance is associated with upregulated pro-inflammatory fatty acids in human cell membranes. Clin Nutr. 2010;29(3):386–90. doi: 10.1016/j.clnu.2009.09.006 19875212

[21] Hoffmann SW, Schierbauer J, Zimmermann P, Voit T, Grothoff A, Wachsmuth NB, et al. Effects of Interrupting Prolonged Sitting with Light-Intensity Physical Activity on Inflammatory and Cardiometabolic Risk Markers in Young Adults with Overweight and Obesity: Secondary Outcome Analyses of the SED-ACT Randomized Controlled Crossover Trial. Biomolecules. 2024;14(8):1029. doi: 10.3390/biom14081029 39199416 PMC11352707

[22] Aggarwal R, Yeh RW, Joynt Maddox KE, Wadhera RK. Cardiovascular Risk Factor Prevalence, Treatment, and Control in US Adults Aged 20 to 44 Years, 2009 to March 2020. JAMA. 2023;329(11):899–909. doi: 10.1001/jama.2023.2307 36871237 PMC9986841

[23] Wong ND, Sattar N. Cardiovascular risk in diabetes mellitus: epidemiology, assessment and prevention. Nat Rev Cardiol. 2023;20(10):685–95. doi: 10.1038/s41569-023-00877-z 37193856

[24] Misra S, Ke C, Srinivasan S, Goyal A, Nyriyenda MJ, Florez JC, et al. Current insights and emerging trends in early-onset type 2 diabetes. Lancet Diabetes Endocrinol. 2023;11(10):768–82. doi: 10.1016/S2213-8587(23)00225-5 37708901

[25] Hill MA, Yang Y, Zhang L, Sun Z, Jia G, Parrish AR, et al. Insulin resistance, cardiovascular stiffening and cardiovascular disease. Metabolism. 2021;119:154766. doi: 10.1016/j.metabol.2021.154766 33766485

[26] Shoelson SE, Lee J, Goldfine AB. Inflammation and insulin resistance. J Clin Invest. 2006;116(7):1793–801. doi: 10.1172/JCI29069 16823477 PMC1483173

[27] Olefsky JM, Glass CK. Macrophages, inflammation, and insulin resistance. Annu Rev Physiol. 2010;72:219–46. doi: 10.1146/annurev-physiol-021909-135846 20148674

[28] Suren Garg S, Kushwaha K, Dubey R, Gupta J. Association between obesity, inflammation and insulin resistance: Insights into signaling pathways and therapeutic interventions. Diabetes Res Clin Pract. 2023;200:110691. doi: 10.1016/j.diabres.2023.110691 37150407

[29] Artunc F, Schleicher E, Weigert C, Fritsche A, Stefan N, Häring H-U. The impact of insulin resistance on the kidney and vasculature. Nat Rev Nephrol. 2016;12(12):721–37. doi: 10.1038/nrneph.2016.145 27748389

[30] Legedz L, Bricca G, Lantelme P, Rial M-O, Champomier P, Vincent M, et al. Insulin resistance and plasma triglyceride level are differently related to cardiac hypertrophy and arterial stiffening in hypertensive subjects. Vasc Health Risk Manag. 2006;2(4):485–90. doi: 10.2147/vhrm.2006.2.4.485 17323603 PMC1994018

[31] Manco M, Grugni G, Di Pietro M, Balsamo A, Di Candia S, Morino GS, et al. Triglycerides-to-HDL cholesterol ratio as screening tool for impaired glucose tolerance in obese children and adolescents. Acta Diabetol. 2016;53(3):493–8. doi: 10.1007/s00592-015-0824-y 26687197

[32] Eeg-Olofsson K, Gudbjörnsdottir S, Eliasson B, Zethelius B, Cederholm J, NDR. The triglycerides-to-HDL-cholesterol ratio and cardiovascular disease risk in obese patients with type 2 diabetes: an observational study from the Swedish National Diabetes Register (NDR). Diabetes Res Clin Pract. 2014;106(1):136–44. doi: 10.1016/j.diabres.2014.07.010 25108897

[33] Qiu Y, Yi Q, Li S, Sun W, Ren Z, Shen Y, et al. Transition of cardiometabolic status and the risk of type 2 diabetes mellitus among middle-aged and older Chinese: A national cohort study. J Diabetes Investig. 2022;13(8):1426–37. doi: 10.1111/jdi.13805 35426487 PMC9340876

[34] Carvalho RL, Brito TRP, Amaral JB, Monteiro FR, Lima DB, Pereira TAM, et al. Unraveling the Interaction between Inflammation and the Cardiometabolic Index in Older Men: A Pilot Study. Nutrients. 2024;16(15):2529. doi: 10.3390/nu16152529 39125408 PMC11313730

[35] Xu B, Wu Q, La R, Lu L, Abdu FA, Yin G, et al. Is systemic inflammation a missing link between cardiometabolic index with mortality? Evidence from a large population-based study. Cardiovasc Diabetol. 2024;23(1):212. doi: 10.1186/s12933-024-02251-w 38902748 PMC11191290

[36] Zou J, Xiong H, Zhang H, Hu C, Lu S, Zou Y. Association between the cardiometabolic index and non-alcoholic fatty liver disease: insights from a general population. BMC Gastroenterol. 2022;22(1):20. doi: 10.1186/s12876-022-02099-y 35021995 PMC8756663

[37] Zha F, Cao C, Hong M, Hou H, Zhang Q, Tang B, et al. The nonlinear correlation between the cardiometabolic index and the risk of diabetes: A retrospective Japanese cohort study. Front Endocrinol (Lausanne). 2023;14:1120277. doi: 10.3389/fendo.2023.1120277 36875460 PMC9980900

[38] Park S, Shimokawa I. Influence of Adipokines on Metabolic Dysfunction and Aging. Biomedicines. 2024;12(4):873. doi: 10.3390/biomedicines12040873 38672227 PMC11048512

[39] Banerjee D, Mani A. Obesity’s systemic impact: exploring molecular and physiological links to diabetes, cardiovascular disease, and heart failure. Front Endocrinol (Lausanne). 2025;16:1681766. doi: 10.3389/fendo.2025.1681766 41282294 PMC12634369

[40] Malavazos AE, Corsi MM, Ermetici F, Coman C, Sardanelli F, Rossi A, et al. Proinflammatory cytokines and cardiac abnormalities in uncomplicated obesity: relationship with abdominal fat deposition. Nutr Metab Cardiovasc Dis. 2007;17(4):294–302. doi: 10.1016/j.numecd.2006.01.001 17434052

[41] Selvaraj S, Martinez EE, Aguilar FG, Kim K-YA, Peng J, Sha J, et al. Association of Central Adiposity With Adverse Cardiac Mechanics: Findings From the Hypertension Genetic Epidemiology Network Study. Circ Cardiovasc Imaging. 2016;9(6):10.1161/CIRCIMAGING.115.004396 e004396. doi: 10.1161/CIRCIMAGING.115.004396 27307550 PMC4911824

[42] Ho JE, McCabe EL, Wang TJ, Larson MG, Levy D, Tsao C, et al. Cardiometabolic Traits and Systolic Mechanics in the Community. Circ Heart Fail. 2017;10(5):e003536. doi: 10.1161/CIRCHEARTFAILURE.116.003536 28495953 PMC5500189

[43] Schouten F, Twisk JW, de Boer MR, Stehouwer CD, Serné EH, Smulders YM, et al. Increases in central fat mass and decreases in peripheral fat mass are associated with accelerated arterial stiffening in healthy adults: the Amsterdam Growth and Health Longitudinal Study. Am J Clin Nutr. 2011;94(1):40–8. doi: 10.3945/ajcn.111.013532 21562083

[44] Raheem J, Sliz E, Shin J, Holmes MV, Pike GB, Richer L, et al. Visceral adiposity is associated with metabolic profiles predictive of type 2 diabetes and myocardial infarction. Commun Med (Lond). 2022;2:81. doi: 10.1038/s43856-022-00140-5 35789567 PMC9249739

[45] Alderete TL, Toledo-Corral CM, Desai P, Weigensberg MJ, Goran MI. Liver fat has a stronger association with risk factors for type 2 diabetes in African-American compared with Hispanic adolescents. J Clin Endocrinol Metab. 2013;98(9):3748–54. doi: 10.1210/jc.2013-1138 23873990 PMC3763973

[46] Zhong P, Tan S, Zhu Z, Liang Y, Huang W, Wang W. Normal-weight central obesity and risk of cardiovascular and microvascular events in adults with prediabetes or diabetes: Chinese and British cohorts. Diabetes Metab Res Rev. 2023;39(8):e3707. doi: 10.1002/dmrr.3707 37525502

[47] Lechner K, Lechner B, Crispin A, Schwarz PEH, von Bibra H. Waist-to-height ratio and metabolic phenotype compared to the Matsuda index for the prediction of insulin resistance. Sci Rep. 2021;11(1):8224. doi: 10.1038/s41598-021-87266-z 33859227 PMC8050044

[48] Bai J, Zhang Y, He L, Zhao Y. Normal Weight Central Obesity and its Impact on Type 2 Diabetes Mellitus. Curr Diab Rep. 2024;25(1):3. doi: 10.1007/s11892-024-01559-x 39503788

[49] Nakashima R, Ikeda S, Shinohara K, Matsumoto S, Yoshida D, Ono Y, et al. Triglyceride/high density lipoprotein cholesterol index and future cardiovascular events in diabetic patients without known cardiovascular disease. Sci Rep. 2025;15(1):9217. doi: 10.1038/s41598-025-92933-6 40097497 PMC11914472

