# Supplementary material for: Impact of cardiometabolic index on long-term mortality in young adults with type 2 diabetes mellitus
Source: PLoS One. 2026 May 21;21(5):e0348952. doi: 10.1371/journal.pone.0348952 (PMC13193537; doi:10.1371/journal.pone.0348952)
Supplement: S1 Fig — (PDF) [file pone.0348952.s001.pdf]

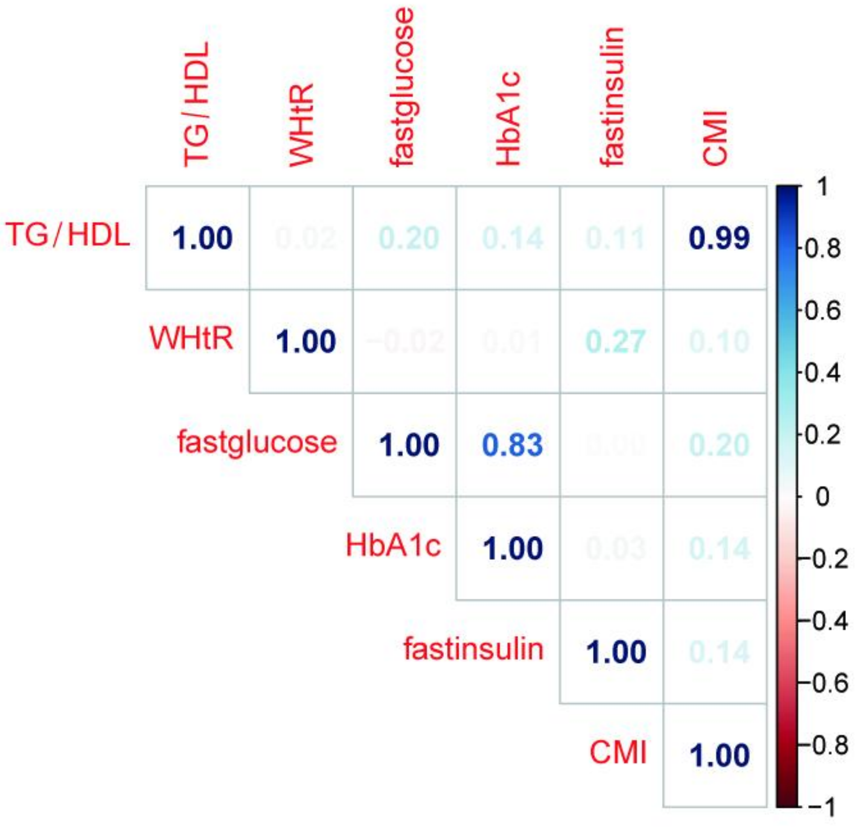

S1 Fig. The Spearman correlation coefficients among CMI, TG/HDL, WHtR, fast glucose, HbA1c, and fast insulin.

Abbreviations: CMI: cardiometabolic index; WHtR: waist-to-height ratio; TG: triglyceride; HDL: high-density lipoprotein cholesterol; HbA1c: glycated hemoglobin A1c.
