# Supplementary material for: Impact of cardiometabolic index on long-term mortality in young adults with type 2 diabetes mellitus
Source: PLoS One. 2026 May 21;21(5):e0348952. doi: 10.1371/journal.pone.0348952 (PMC13193537; doi:10.1371/journal.pone.0348952)
Supplement: S2 Fig — (PDF) [file pone.0348952.s002.pdf]

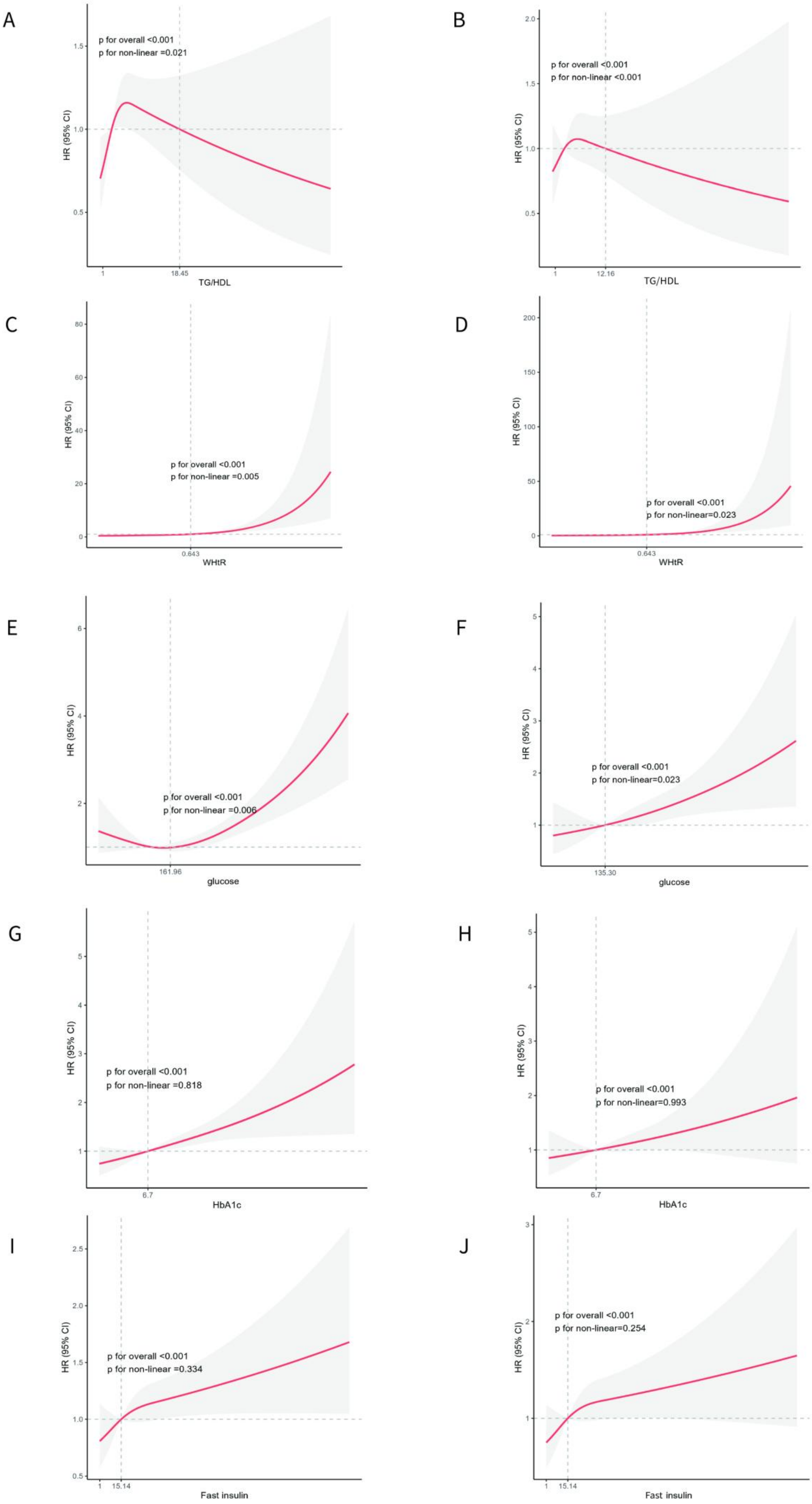

A
p for overall <0.001
p for non-linear =0.021
HR (95% CI)
TG/HDL
18.45
B
p for overall <0.001
p for non-linear <0.001
HR (95% CI)
TG/HDL
12.16
C
p for overall <0.001
p for non-linear =0.005
HR (95% CI)
WHtR
0.643
D
p for overall <0.001
p for non-linear=0.023
HR (95% CI)
WHtR
0.643
E
p for overall <0.001
p for non-linear =0.006
HR (95% CI)
glucose
161.96
F
p for overall <0.001
p for non-linear=0.023
HR (95% CI)
glucose
135.30
G
p for overall <0.001
p for non-linear =0.818
HR (95% CI)
HbA1c
6.7
H
p for overall <0.001
p for non-linear=0.993
HR (95% CI)
HbA1c
6.7
I
p for overall <0.001
p for non-linear =0.334
HR (95% CI)
Fast insulin
15.14
J
p for overall <0.001
p for non-linear=0.254
HR (95% CI)
Fast insulin
15.14

S2 Fig. The Restricted cubic spline analysis between TG/HDL, WHtR, fast glucose, HbA1c, fast insulin and the risk of all-cause mortality and CVD mortality in participants with diabetes.

(A) TG/HDL and the risk of all-cause mortality; (B) TG/HDL and the risk of CVD mortality; (C) WHtR and the risk of all-cause mortality; (D) WHtR and the risk of CVD mortality; (E) fast glucose and the risk of all-cause mortality; (F) fast glucose and the risk of CVD mortality; (G) HbA1c and the risk of all-cause mortality; (H) HbA1c and the risk of CVD mortality; (I) fast insulin and the risk of all-cause mortality; (J) fast insulin and the risk of CVD mortality.

Abbreviations: WHtR: waist-to-height ratio; TG: triglyceride; HDL: high-density lipoprotein cholesterol; HbA1c: glycated hemoglobin A1c; CVD: cardiovascular disease.
