# Supplementary material for: Impact of cardiometabolic index on long-term mortality in young adults with type 2 diabetes mellitus
Source: PLoS One. 2026 May 21;21(5):e0348952. doi: 10.1371/journal.pone.0348952 (PMC13193537; doi:10.1371/journal.pone.0348952)
Supplement: S3 Fig — (PDF) [file pone.0348952.s003.pdf]

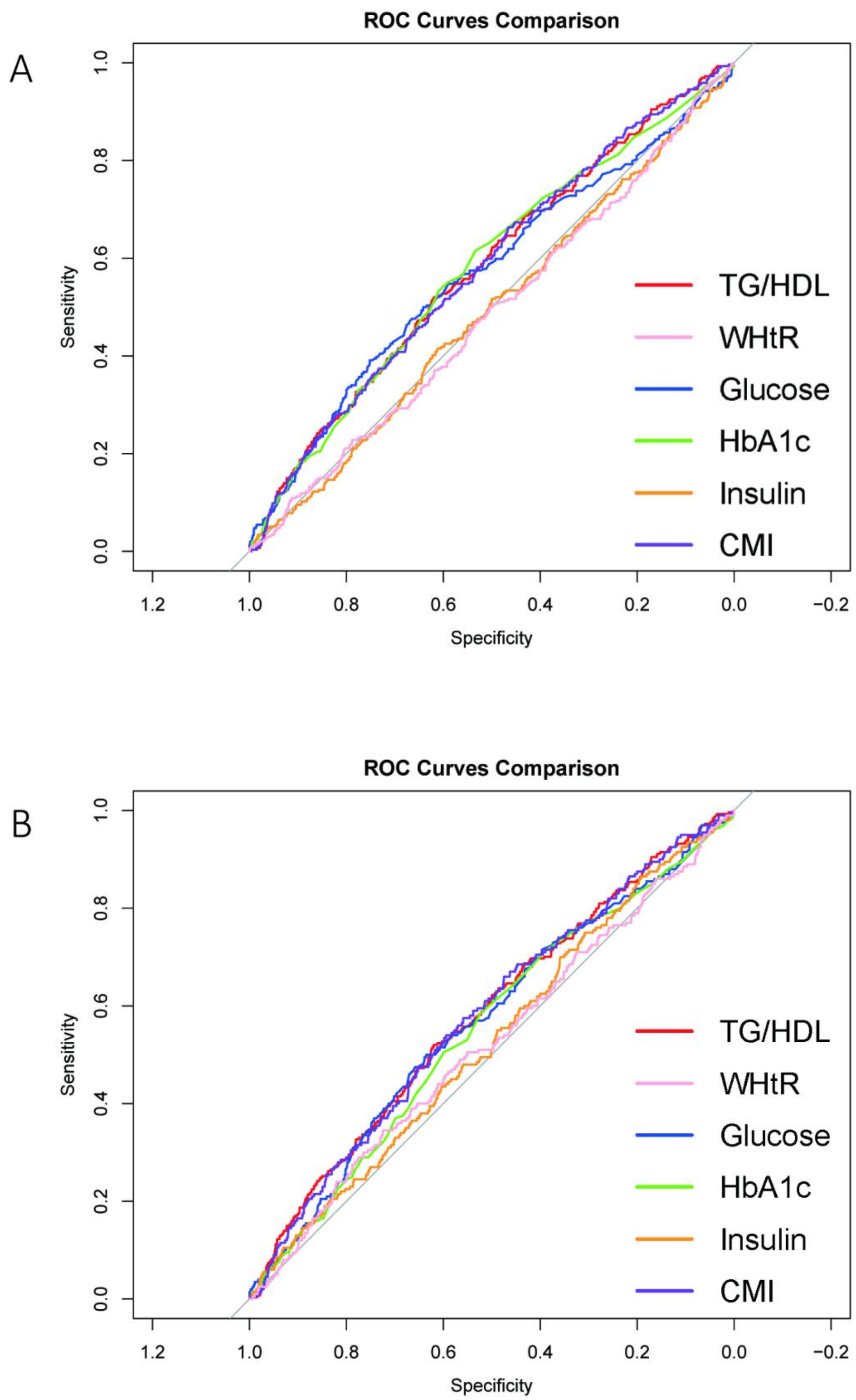

S3 Fig. Receiver operating characteristic (ROC) curves of CMI and other metabolic indicators for predicting mortality risk. Receiver operating characteristic (ROC) curves of CMI, TG/HDL, WHtR, fasting glucose, HbA1c, and fasting insulin for the risk of all-cause mortality (A) and CVD mortality (B) in participants with diabetes.

Abbreviations: CMI: cardiometabolic index; WHtR: waist-to-height ratio; TG: triglyceride; HDL: high-density lipoprotein cholesterol; HbA1c: glycated hemoglobin A1c; CVD: cardiovascular disease.
