# Supplementary material for: Impact of cardiometabolic index on long-term mortality in young adults with type 2 diabetes mellitus
Source: PLoS One. 2026 May 21;21(5):e0348952. doi: 10.1371/journal.pone.0348952 (PMC13193537; doi:10.1371/journal.pone.0348952)
Supplement: S4 Fig — (PDF) [file pone.0348952.s004.pdf]

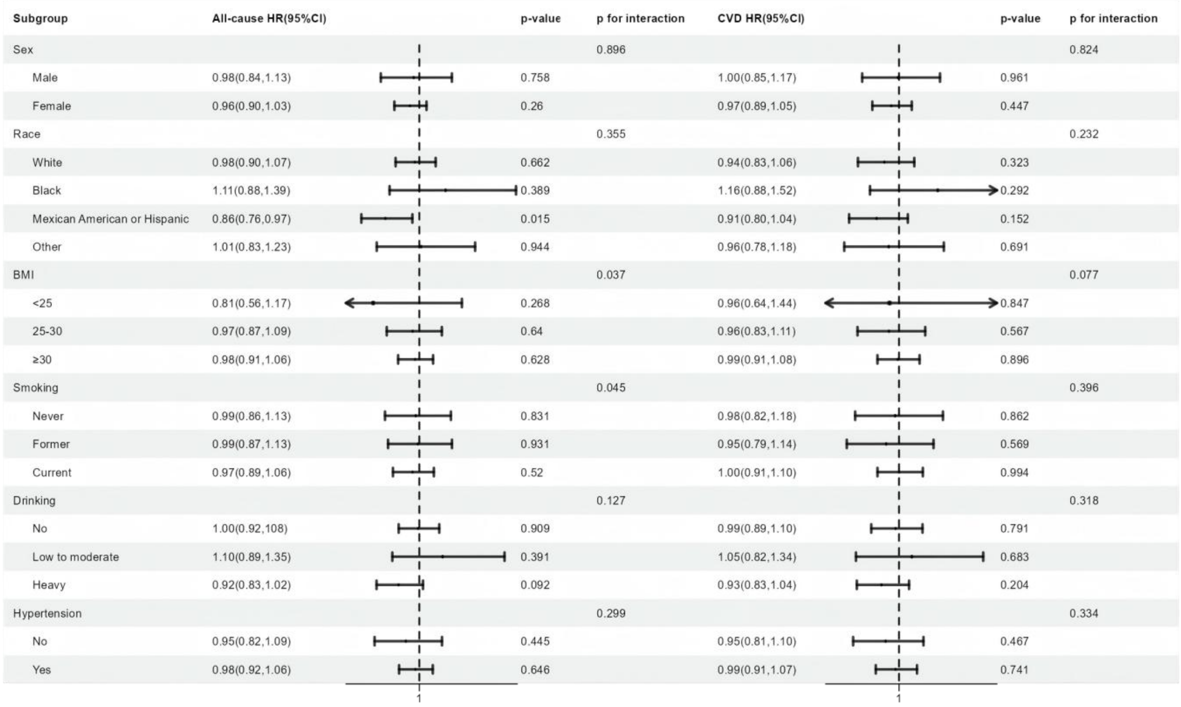

S4 Fig. Stratified analyses of the associations between TG/HDL and all-cause mortality and CVD mortality among young individuals with diabetes.

Abbreviation: TG: triglyceride; HDL: high-density lipoprotein cholesterol; BMI: body mass index; CVD: cardiovascular disease.
