# Supplementary material for: Impact of cardiometabolic index on long-term mortality in young adults with type 2 diabetes mellitus
Source: PLoS One. 2026 May 21;21(5):e0348952. doi: 10.1371/journal.pone.0348952 (PMC13193537; doi:10.1371/journal.pone.0348952)
Supplement: S1 Table — (PDF) [file pone.0348952.s005.pdf]

Supplementary Materials

Impact of Cardiometabolic Index on Long-Term Mortality in Young Adults with Type 2 Diabetes Mellitus

Running title: Cardiometabolic Index and Mortality in Young Adults with T2DM

S1 Table. Association between TG/HDL, WHtR, glucose, HbA1c, fast insulin and all-cause mortality and CVD mortality in T2DM populations.

	Model 1		Model 2		Model 3	
	HR(95%CI)	*p*-value	HR(95%CI)	*p*-value	HR(95%CI)	*p*-value
**All-cause mortality**						
**TG/HDL**	1.004(0.993, 1.015)	0.498	1.004(0.992, 1.017)	0.524	0.995(0.980, 1.010)	0.696
**WHtR**	3.951(0.987, 15.820)	0.052	5.159(0.990, 26.891)	0.051	2.610(0.712, 5.012)	0.104
**glucose**	1.003(1.002,1.005)	<0.001	1.004(1.002, 1.006)	<0.001	1.004(1.003, 1.006)	<0.001
**HbA1c**	1.099(1.033, 1.168)	0.003	1.146(1.069, 1.230)	<0.001	1.142(1.061, 1.229)	<0.001
**Fast insulin**	1.004(1.001, 1.006)	0.005	1.003(0.999, 1.006)	0.091	1.003(1.000, 1.006)	0.033
**CVD mortality**						
**TG/HDL**	1.001(0.986,1.015)	0.918	0.999(0.984,1.014)	0.889	0.994(0.977, 1.011)	0.457
**WHtR**	4.807(0.864, 26.770)	0.073	6.360(0.874, 46.279)	0.068	2.292(0.583, 5.021)	0.102
**glucose**	1.003(1.001, 1.005)	0.004	1.004(1.002, 1.006)	0.001	1.003(1.001, 1.006)	0.003
**HbA1c**	1.053(0.973, 1.140)	0.199	1.087(0.994, 1.190)	0.068	1.085(0.990, 1.188)	0.081
**Fast insulin**	1.004(1.001, 1.006)	0.008	1.003(1.000, 1.006)	0.088	1.003(1.000, 1.006)	0.069

Abbreviations: WHtR: waist-to-height ratio; TG: triglyceride; HDL: high-density lipoprotein cholesterol; HbA1c: glycated hemoglobin A1c; CVD: cardiovascular disease.
