# Supplementary material for: Impact of cardiometabolic index on long-term mortality in young adults with type 2 diabetes mellitus
Source: PLoS One. 2026 May 21;21(5):e0348952. doi: 10.1371/journal.pone.0348952 (PMC13193537; doi:10.1371/journal.pone.0348952)
Supplement: S2 Table — (PDF) [file pone.0348952.s006.pdf]

S2 Table. AUC of TG/HDL, WHtR, glucose, HbA1c, fast insulin and all-cause mortality and CVD mortality in T2DM populations.

	ROC	
	AUC	*p*-value
**All-cause mortality**		
**CMI**	0.579	Reference
**TG/HDL**	0.581	0.689
**WHtR**	0.485	<0.001
**glucose**	0.568	0.650
**HbA1c**	0.579	0.996
**Fast insulin**	0.491	0.004
**CVD mortality**		
**CMI**	0.579	Reference
**TG/HDL**	0.581	0.948
**WHtR**	0.521	0.017
**glucose**	0.566	0.636
**HbA1c**	0.552	0.019
**Fast insulin**	0.526	0.008

Abbreviations: CMI: cardiometabolic index; WHtR: waist-to-height ratio; TG: triglyceride; HDL: high-density lipoprotein cholesterol; HbA1c: glycated hemoglobin A1c; CVD: cardiovascular disease.
